# TRAF3 regulates STAT6 activation and T-helper cell differentiation by modulating the phosphatase PTP1B

**DOI:** 10.1016/j.jbc.2024.107737

**Published:** 2024-09-02

**Authors:** Tina Arkee, Emma L. Hornick, Gail A. Bishop

**Affiliations:** 1 Department of Microbiology and Immunology, The University of Iowa, Iowa City, Iowa, USA; 2 Medical Scientist Training Program, The University of Iowa, Iowa City, Iowa, USA; 3 Department of Internal Medicine, The University of Iowa, Iowa City, Iowa, USA; 4 Office of Research and Development, Iowa City VA Medical Center, Iowa City, Iowa, USA

**Keywords:** adaptor, TRAF, T cell, signal transduction, IL-4R, STAT, PTP

## Abstract

The adaptor protein tumor necrosis factor receptor–associated factor 3 (TRAF3) is a multifaceted regulator of lymphocyte biology that plays key roles in modulation of the molecular signals required for T-cell activation and function. TRAF3 regulates signals mediated by the T-cell receptor (TCR), costimulatory molecules, and cytokine receptors, which each drive activation of the serine/threonine kinase Akt. The impact of TRAF3 upon TCR–CD28-mediated activation of Akt, and thus on the diverse cellular processes regulated by Akt, including CD4 T-cell fate decisions, remains poorly understood. We show here that TRAF3 deficiency led to impaired Akt activation and thus to impaired *in vitro* skewing of CD4 T cells into the T_H_1 and T_H_2 fates. We investigated the role of TRAF3 in regulation of signaling pathways that drive T_H_1 and T_H_2 differentiation and found that TRAF3 enhanced activation of signal transducer and activator of transcription 6 (STAT6), thus promoting skewing toward the T_H_2 fate. TRAF3 promoted STAT6 activation by regulating recruitment of the inhibitory molecule protein tyrosine phosphatase 1B to the IL-4R signaling complex, in a manner that required integration of TCR–CD28- and IL-4R-mediated signals. This work reveals a new mechanism for TRAF3-mediated regulation of STAT6 activation in CD4 T cells and adds to our understanding of the diverse roles played by TRAF3 as an important regulator of T-cell biology.

The intracellular adaptor protein tumor necrosis factor receptor–associated factor 3 (TRAF3) plays diverse cell type- and context-specific roles as a multifaceted regulator of lymphocyte biology (1). TRAF3 impacts the homeostasis and function of T-cell subsets by regulating the molecular signals that drive T-cell activation, including signals mediated by the T-cell receptor (TCR), costimulatory molecules, and cytokine receptors (reviewed in Refs. (2, 3)). In the absence of TRAF3, altered recruitment of inhibitory molecules such as the tyrosine kinase c-Src kinase (Csk), and the tyrosine phosphatases protein tyrosine phosphatase (PTP) PTPN22 and PTP1B, contributes to markedly defective TCR–CD28 signaling (4, 5), which subsequently impacts T-cell proliferation, cytokine production, and function in complex *in vivo* immune responses (6).

TCR- and CD28-mediated signals promote activation of the serine/threonine kinase Akt (also known as protein kinase B). Akt, in turn, acts as a key regulator of diverse cellular processes, including metabolism, proliferation, and differentiation, through modulation of mammalian target of rapamycin (mTOR) signaling (7, 8, 9). Interestingly, TRAF3 deficiency does not impact Akt activation by IL-2R- and glucocorticoid-induced tumor necrosis factor receptor–mediated signaling (10, 11). The impact of TRAF3 upon TCR–CD28-mediated Akt activation, and the potential consequences for T-cell fate decisions, was unknown prior to the present study, and the context-dependent nature of TRAF3-mediated regulation, which varies by both cell type and individual receptors in the same cells, makes this an important knowledge gap.

Early studies in CD4^Cre^*Traf3*^flox/flox^ (T-*Traf3*^−/−^) mice provided the first hints of a potential role for TRAF3 in regulation of T-cell fate. These mice have a two- to threefold increase in the frequency and number of thymic regulatory T cells (Treg). This is in part the result of loss of restraint of IL-2R signaling, as TRAF3 is required for recruitment of the phosphatase T-cell PTP/PTPN2 to the IL-2R complex (6, 10). Invariant natural killer T-cell (iNKT) development is impaired in T-*Traf3*^−/−^ mice due to a failure of precursor cells to upregulate the transcription factor T-box expressed in T cells (T-bet) upon TCR stimulation (12). Cell-mediated immune responses are defective in T-*Traf3*^*−/−*^ mice challenged with the intracellular pathogen *Listeria monocytogenes*, and activated TRAF3-deficient CD4 T cells produce reduced amounts of IFNɣ (important for T_H_1 differentiation) and IL-4 (important for T_H_2 differentiation) (6). Human patients with *TRAF3* haploinsufficiency have increased proportions of Treg and circulating T follicular helper (T_FH_) cells and a phenotype of immunodeficiency and autoimmunity that is in part due to dysregulated T-cell help. Activated CD4 T cells from these patients produce reduced amounts of IFNɣ and IL-17 (important for T_H_17 differentiation) (13). Thus, TRAF3 plays an important role in differentiation and function of CD4 T-cell subsets. However, the precise molecular mechanisms by which TRAF3 regulates these processes are not clear.

There is an established role for T-cell TRAF3 in modulation of signaling mediated by the TCR (4, 6), costimulatory receptors (5, 11), and the cytokine receptors IL-2R and interferon (IFN) alpha and beta receptor (10, 14). There is also emerging evidence in support of a role for T-cell TRAF3 in regulation of IL-6R signaling (15). However, the role of TRAF3 in signaling mediated by IFNɣR and IL-4R is poorly understood. These cytokine receptors promote activation of the transcription factors STAT1 and STAT6, respectively, which in turn drive expression of the lineage-defining transcription factors T-bet and GATA-binding protein 3 (GATA3). As there was precedent for TRAF3 regulating T-bet in iNKT cells (12) and STAT6 in macrophages (16), it was of considerable interest to understand the impact of TRAF3 upon integration of signals from the TCR, CD28, and cytokine receptors on downstream signaling.

Results presented here show that the defective TCR–CD28 signaling seen in the absence of TRAF3 led to impaired activation of Akt, with subsequent impairment of *in vitro* skewing of CD4 T cells toward the T_H_1 and T_H_2 fates, indicating that TRAF3 regulates signaling pathways underlying CD4 T-cell fate decisions. We found that TRAF3 enhanced STAT6 activation by regulating recruitment of the phosphatase PTP1B in a manner that requires integration of TCR–CD28- and IL-4R-mediated signals. These results reveal a new mechanism by which STAT6 activation is modulated by TRAF3 and add to our growing understanding of the diverse roles played by TRAF3 as a multifaceted regulator of T-cell biology.

## Results

### Impact of TRAF3 upon early TCR–CD28-mediated Akt activation

TRAF3 is recruited to the TCR–CD28 complex upon engagement of both CD3 and CD28 (6), enhancing TCR–CD28-mediated signaling *via* restraint of the negative regulatory molecules Csk, PTPN22, and Dok1 (4, 5). TCR- and CD28-mediated signaling lead to the activation of multiple signaling pathways important for T-cell function, including the PI3K–Akt–mTOR pathway, which plays an important role in regulation of CD4 T-cell differentiation (7, 9, 17). Downstream of PI3K activation, Akt is activated by phosphorylation at T308. Further phosphorylation of Akt at S473 confers optimal activation. Phosphorylation of these two residues provides insight into mTOR signaling: phosphorylation of Akt at T308 is upstream of mTORC1, whereas phosphorylation of Akt at S473 is mediated by mTORC2 (9). mTOR signaling through mTORC1 *versus* mTORC2 impacts CD4 T-cell fate decisions (7, 18, 19, 20). Given our previous findings that TRAF3 enhances TCR proximal signaling (6), we wished to determine how TRAF3 impacted Akt activation in CD4 T cells. We thus assessed early TCR–CD28-mediated activation of the serine/threonine kinase Akt.

**Figure 1:**
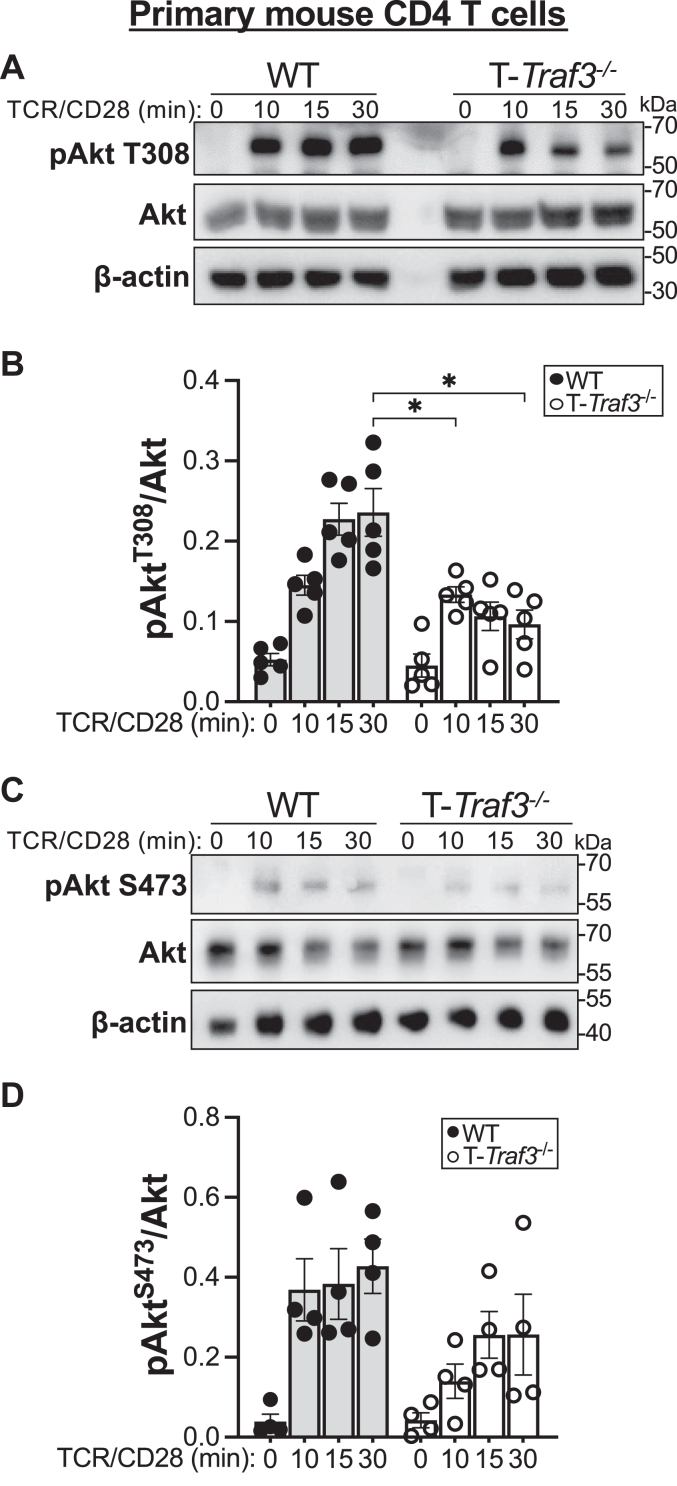
**Impact of TRAF3 upon TCR–CD28-mediated Akt activation.** Primary mouse CD4 T cells were stimulated with mAbs against CD3 and CD28 (“TCR–CD28”) for the indicated number of minutes, lysed, and indicated proteins were quantified by Western blotting. *A*, representative Western blot of phospho-Akt T308 and total Akt. *B*, quantification of four biological replicates (each symbol corresponds to one replicate), including blot shown in (*A*). *C*, representative Western blot of phospho-Akt S473 and total Akt in mouse CD4 T cells following TCR–CD28 stimulation. *D*, quantification of four biological replicates (each symbol corresponds to one replicate), including blot shown in (*C*). ∗*p* < 0.05 by Sidak multiple comparisons test. Error bars represent SEM. mAb, monoclonal antibody; TCR, T-cell receptor; TRAF3; tumor necrosis factor receptor–associated factor 3.

We predicted that TCR–CD28-mediated Akt phosphorylation at both T308 and S473 would be diminished in TRAF3-deficient primary mouse CD4 T cells. WT cells had significantly more phospho-Akt T308 than TRAF3-deficient cells 30 min after TCR–CD28 stimulation (Fig. 1, *A* and *B*). Phospho-Akt T308 reaches its maximum in TRAF3-deficient cells at 10 min but increases in WT cells through 30 min. Even at their respective peaks, TRAF3-deficient T cells have significantly less phospho-Akt T308 than WT T cells (Fig. 1, *A* and *B*).

Phosphorylation of Akt at S473 showed a trending decrease in TRAF3-deficient T cells compared with WT T cells following TCR–CD28 stimulation, though the difference was not statistically significant (Fig. 1, *C* and *D*). This impairment in Akt phosphorylation, together with evidence supporting a role for differential Akt activation in CD4 T-cell differentiation (18, 19, 20, 21), led us to investigate the role of TRAF3 in *in vitro* CD4 T-cell polarization.

### Role of TRAF3 in *in vitro* polarization of T_H_1 and T_H_2 cells

We previously reported reduced production of IL-2, IFNɣ, and TNFɑ by TRAF3-deficient CD4 T cells cultured under T_H_1-polarizing conditions (6) but did not examine the proportion of TRAF3-deficient T cells expressing the T_H_1 master regulator T-bet relative to their WT counterparts. There is an established role for crossregulation of T_H_1 differentiation by T_H_2 cytokines and the T_H_2 master regulator GATA3 (22, 23, 24). We thus tested the ability of mature CD4 T cells to differentiate into both T_H_1 and T_H_2 cells under both the appropriate *in vitro* polarizing conditions and in the presence of only stimulatory antibodies (Abs) against CD3 and CD28, indicated as “TCR–CD28.”

**Figure 2:**
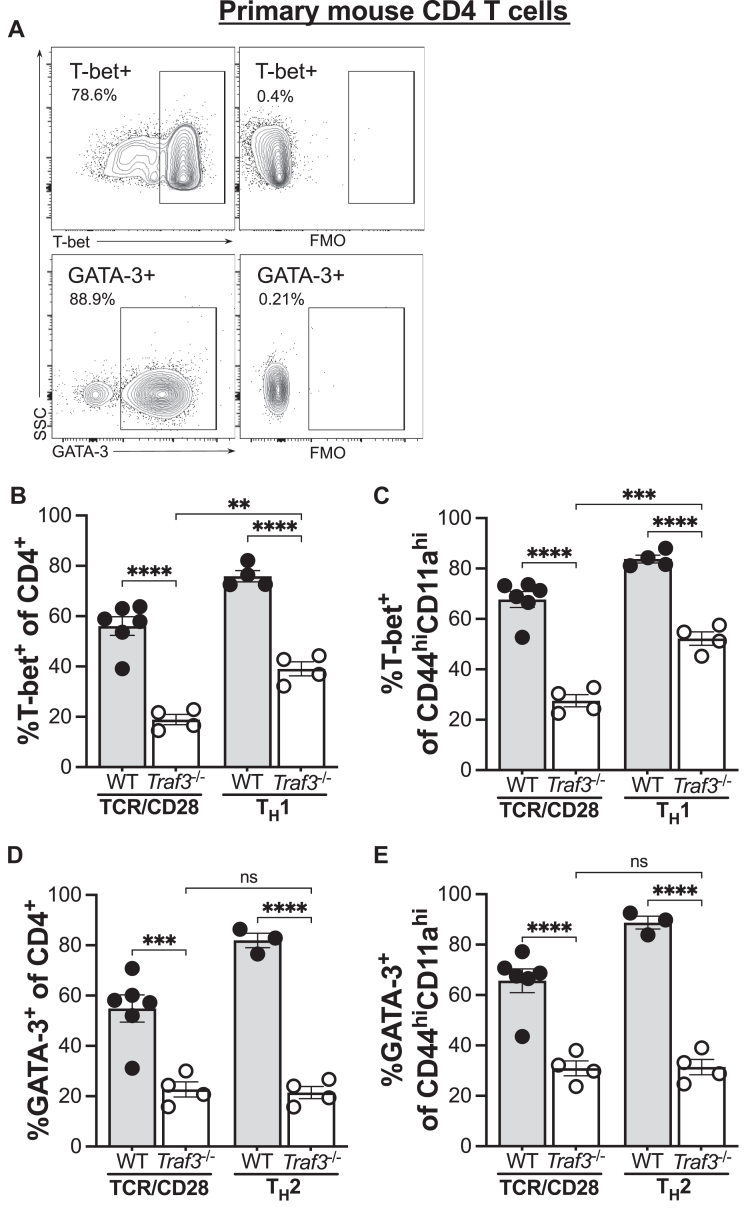
**Effect of TRAF3 on *in vitro* T**_**H**_**polarization.** Primary mouse CD4 T cells were cultured with mAbs against CD3 and CD28 (“TCR–CD28”) or under T_H_1- or T_H_2 polarizing conditions as detailed in *Experimental procedures* section. Abundance of the indicated surface and intracellular proteins was assessed by flow cytometry. Each symbol represents one biological replicate. *A*, flow cytometry gating strategy. *B* and *C*, frequency of T-bet^+^ cells among total CD4^+^ T cells (*B*) and antigen-experienced CD4^+^ T cells (*C*). *D* and *E*, frequency of GATA3^+^ cells among total CD4^+^ T cells (*D*) and antigen-experienced CD4^+^ T cells (*E*). ∗∗*p* < 0.001, ∗∗∗*p* < 0.001, and ∗∗∗∗*p* < 0.0001 by Sidak multiple comparisons test. Error bars represent SEM. GATA3, GATA-binding protein 3; mAb, monoclonal antibody; TRAF3, tumor necrosis factor receptor–associated factor 3.

Upregulation of the T_H_1-associated transcription factor T-bet during T_H_ subset differentiation is driven by IFNɣ-mediated activation of STAT1 and IL-12-mediated activation of STAT4 (24). We previously reported that TRAF3-deficient CD4 T cells have reduced production of IL-2, IFNɣ, and TNFɑ under T_H_1-polarizing conditions (6) and that TRAF3-deficient iNKT cells have decreased T-bet expression (12). Consistent with this, there was a decreased proportion of T-bet^+^ TRAF3-deficient T cells compared with their WT counterparts cultured in the presence of Abs against CD3 and CD28 alone (Fig. 2, *A*–*C*). The proportion of T-bet^+^ TRAF3-deficient T cells increased under T_H_1-polarizing conditions compared with TCR–CD28 stimulation condition (Fig. 2, *A*–*C*) but did not reach WT levels, suggesting that there was a partial response to T_H_1 cytokines.

The proportion of GATA3^+^ TRAF3-deficient CD4 T cells was decreased compared with WT CD4 T cells cultured with TCR–CD28 or with T_H_2 polarizing conditions (Fig. 2, *D* and *E*). Importantly, T_H_2 polarization conditions were no more effective than TCR–CD28 stimulation alone at inducing GATA3 in TRAF3-deficient CD4 T cells, in contrast to an increase in GATA3^+^ WT CD4 T cells under T_H_2 polarization conditions (Fig. 2, *D* and *E*). These data suggested an additional defect in the ability of TRAF3-deficient T cells to respond to a signal unique to the T_H_2-polarizing cocktail, which includes IL-4 and an IFNɣ blocking Ab in addition to TCR–CD28. We thus considered the possibility that altered IL-4R signaling in TRAF3-deficient CD4 T cells impaired T_H_2 differentiation.

### Impact of TRAF3 upon STAT1 activation

Alterations in IFNɣR signaling in TRAF3-deficient T cells could affect T_H_1 differentiation, thus we examined IFNɣR-mediated phosphorylation of STAT1 at Y701, an activating residue. There was a trending increase in the amount of pSTAT1 Y701 relative to total STAT1 in TRAF3-deficient CD4 T cells cultured in the presence of Abs against CD3 and CD28 compared with their WT counterparts, with no appreciable difference in expression of total STAT1 after 24 h of *in vitro* culture (Fig. S1, *A*–*C*). We also examined early IFNɣR signaling and found no difference in IFNɣ-mediated phosphorylation of STAT1 at Y701 (Fig. S1, *D* and *E*), indicating that defects in IFNɣR signaling are not likely responsible for T_H_ polarization defects seen in TRAF3-deficient CD4 T cells.

### STAT6 activation in TRAF3-deficient CD4 T cells

**Figure 3:**
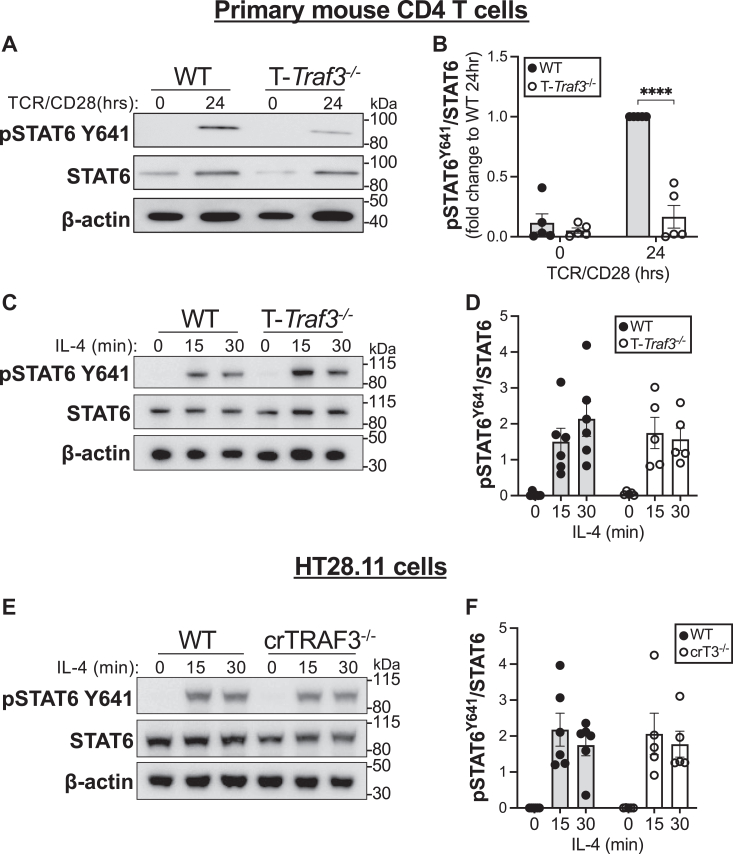
**Impact of TRAF3 on STAT6 activation in T cells.** Primary mouse CD4 T cells or HT28.11 cells were left unstimulated, stimulated through TCR–CD28, or cultured with IL-4 for the indicated number of minutes/hours. Cells were lysed, and indicated proteins were quantified by Western blotting. *A*, representative Western blots of phospho-STAT6 Y641 and total STAT6. *B*, quantification of independent experiments, including blot shown in (*A*). Each symbol represents one biological replicate. *C*, representative Western blots of phospho-STAT6 Y641 and total STAT6 in primary mouse CD4 T cells stimulated with IL-4. *D*, quantification of three independent experiments, including the blots shown in (*C*). Each symbol represents one biological replicate. *E*, representative Western blots of phospho-STAT6 Y641 and total STAT6 in HT28.11 cells stimulated with IL-4. *F*, quantification of six independent replicates, including the blots shown in (*E*). ∗∗∗∗*p* < 0.0001 by Sidak multiple comparisons test. Error bars represent SEM. IL-4, interleukin 4; STAT6, signal transducer and activator of transcription 6; TCR; T-cell receptor; TRAF3, tumor necrosis factor receptor–associated factor 3.

Given the failure of TRAF3-deficient mouse T cells to upregulate GATA3 under T_H_2-polarizing conditions, we investigated early signaling events relevant to T_H_2 differentiation. Consistent with the decreased proportion of GATA3^+^ cells observed in TRAF3-deficient T cells cultured under both nonpolarizing and T_H_2-polarizing conditions (Fig. 2, *D* and *E*), we observed a significant reduction in TCR–CD28-induced phosphorylation of STAT6 at the activating residue Y641 (Fig. 3, *A* and *B*). Although STAT6 activation is mediated by both IL-4R-dependent and -independent mechanisms (25, 26), the failure of TRAF3-deficient T cells to upregulate GATA3 when cultured under T_H_2-polarizing conditions (Fig. 2, *D* and *E*) suggested a defect primarily in IL-4R-mediated STAT6 activation. However, we observed no significant difference in STAT6 activation mediated by IL-4 alone in WT *versus* TRAF3-deficient primary mouse CD4 T cells (Fig. 3, *C* and *D*). We observed similar findings in the human CD4 T-cell line HT28.11 (Fig. 3, *E* and *F*). These results, together with those presented in Figure 2, led us to hypothesize that TRAF3 impacts the ability of T cells to respond to IL-4 stimulation specifically in the context of TCR–CD28-mediated activation.

**Figure 4:**
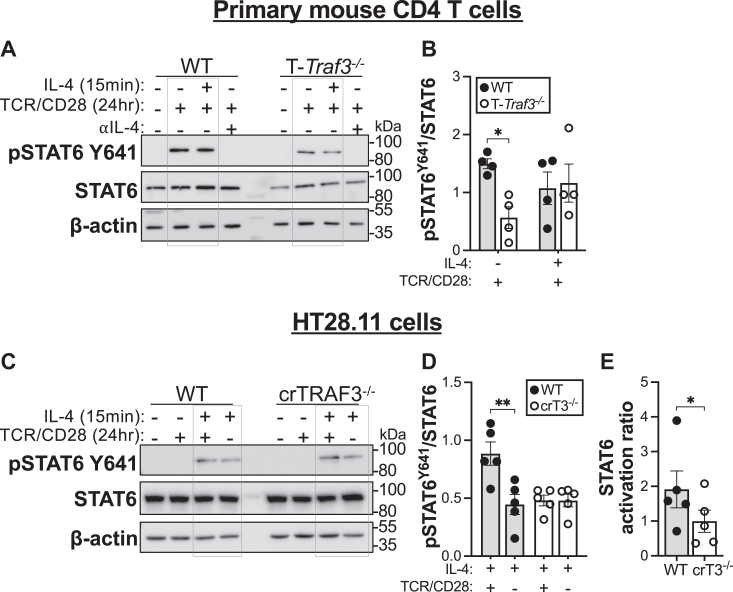
**Impact of TRAF3 on IL-4-mediated STAT6 activation in T cells stimulated through TCR–CD28.** Primary mouse CD4 T cells or HT28.11 cells were stimulated as indicated, then lysed, and the indicated proteins were quantified by Western blotting. *A*, representative Western blots of phospho-STAT6 Y641 and total STAT6 in primary mouse CD4 T cells left untreated or treated with combinations of the following: TCR–CD28 (24 h), IL-4 (during last 15 min of TCR–CD28 stimulation), and an IL-4 neutralizing antibody (24 h). *B*, quantification of four biological replicates, including the blots in (*A*). *C*, representative Western blots of phospho-STAT6 Y641 and total STAT6 in HT28.11 cells left untreated or treated with the indicated combinations of the following: TCR–CD28, 2 h; IL-4, 15 min. *D*, quantification of six independent replicates, including the blots in (*C*). *E*, STAT6 activation ratio (detailed in text) from six independent replicates, including the blots in (*C*). ∗*p* < 0.05, ∗∗*p* < 0.001 by Fisher multiple comparisons test (*B* and *D*) or unpaired *t* test (*E*). Error bars represent SEM. IL-4, interleukin 4; STAT6, signal transducer and activator of transcription 6; TCR; T-cell receptor; TRAF3, tumor necrosis factor receptor–associated factor 3.

To test this hypothesis, we assessed phosphorylation of STAT6 at Y641 in primary mouse CD4 T cells following TCR–CD28 stimulation with or without subsequent IL-4 stimulation. As previously observed, there was a reduction in abundance of pSTAT6 Y641 relative to STAT6 in TRAF3-deficient T cells compared with their WT counterparts (Fig. 4, *A* and *B*). There was no increase in relative pSTAT6 Y641 abundance following restimulation of TCR–CD28-activated cells with IL-4 (Fig. 4, *A* and *B*). We performed a similar experiment in HT28.11 human T cells and found that while TCR–CD28-mediated signaling alone was not sufficient for STAT6 activation, TCR–CD28 signaling enhanced IL-4-mediated STAT6 activation (Fig. 4, *C* and *D*). Consistent with our finding in TRAF3-deficient primary mouse T cells (Fig. 4, *A* and *B*), there was no increase in STAT6 activation in TCR–CD28-activated TRAF3-deficient HT28.11 cells that were restimulated with IL-4 (Fig. 4, *C* and *D*). That is, the amount of STAT6 activation in TRAF3-deficient T cells stimulated through TCR–CD28 + IL-4, when normalized to stimulation with IL-4 alone, was decreased compared with this ratio of STAT6 activation in WT T cells under the same conditions (Fig. 4*E*). Taken together, these results are consistent with our hypothesis that TCR–CD28-mediated signaling *synergizes* with IL-4R signaling to promote STAT6 activation and that this synergistic effect is diminished in TRAF3-deficient T cells, which have impaired TCR–CD28 signaling (5, 6).

### Role of PTP1B in TRAF3-mediated regulation of STAT6

**Figure 5:**
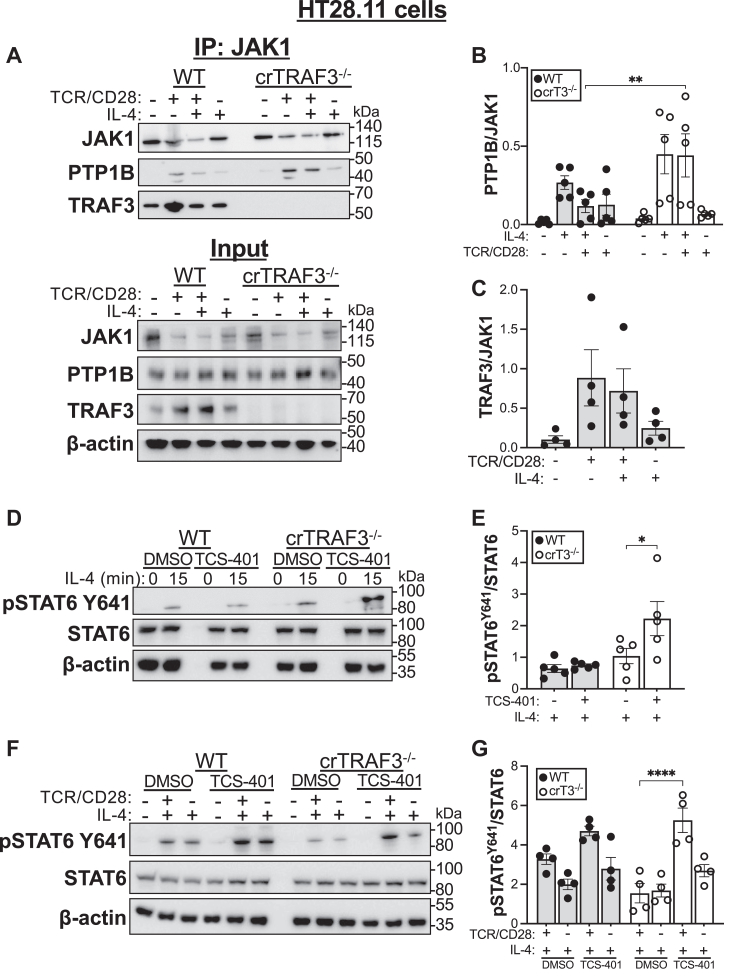
**Role of PTP1B in TRAF3-mediated regulation of STAT6 activation.***A*–*C*, cells of the human CD4 T-cell line HT28.11 were stimulated through TCR–CD28 and/or with IL-4, then JAK1 was immunoprecipitated, and lysates were analyzed by Western blotting. *A*, representative Western blots of JAK1, PTP1B, and TRAF3 coimmunoprecipitating with JAK1 (“IP:JAK1,” *top*) or in whole cell lysates (“input,” *bottom*) from five independent replicates. *B*, quantification of PTP1B coimmunoprecipitating with JAK1 from four independent replicates including the blots in (*A*) (*top*). *C*, quantification of TRAF3 coimmunoprecipitating with JAK1 from four independent replicates including the blots in (*A*) (*top*); only WT HT28.11 samples are quantified because TRAF3 was undetectable in crTRAF3^−/−^ samples. *D*–*G*, cells of the human CD4 T-cell line HT28.11 were treated with the PTP1B inhibitor TCS-401 or an equal volume of dimethyl sulfoxide (DMSO) for 2 h, stimulated through TCR–CD28 and/or with IL-4, then lysed for quantification of proteins of interest by Western blotting. *D*, representative Western blots of phospho-STAT6 Y641 following IL-4 stimulation for the indicated number of minutes with or without TCS-401, as indicated. *E*, quantification of five independent replicates, including the blots shown in (*D*). *F*, representative Western blots of phospho-STAT6 Y641 following TCR–CD28 and/or IL-4 stimulation, ±TCS-401/DMSO, as indicated. *G*, quantification of four independent replicates, including the blots shown in (*F*). ∗*p* < 0.05, ∗∗*p* < 0.01, and ∗∗∗*p* < 0.001 by Tukey multiple comparisons test. Each graphed symbol represents an individual biological replicate, and error bars represent SEM. IL-4, interleukin 4; STAT6, signal transducer and activator of transcription 6; TCR, T-cell receptor; TRAF3, tumor necrosis factor receptor–associated factor 3.

We next focused on how TRAF3 alters IL-4R signaling. PTP PTP1B negatively regulates B-cell IL-4R-mediated STAT6 activation by removing the activating phosphorylation from STAT6 (27, 28, 29). There are many examples of TRAF3 regulating lymphocyte signaling by altering phosphatase localization/association with target proteins; we recently reported that T-cell TRAF3 enhances TCR–CD28-mediated signaling by sequestering PTP1B from its target, the tyrosine kinase breast tumor kinase (5, 30). PTP1B can also act on JAK family members upstream of STAT6 activation (31). Lymphocytes express the type 1 IL-4R complex, which is composed of IL-4Rɑ, ɣ_c_ (IL-2Rɣ), JAK1 (associated with IL-4Rɑ), and JAK3 (associated with ɣ_c_) (32, 33). We predicted that association of PTP1B with JAK1 would be increased in TRAF3-deficient T cells, which have impaired STAT6 activation (Figs. 3, *A* and *B* and 4). Indeed, we saw increased PTP1B–JAK1 association in TRAF3-deficient T cells stimulated through TCR–CD28 with or without IL-4, compared with their WT counterparts (Fig. 5, *A* and *B*). In contrast, there was minimal PTP1B associated with JAK1 in WT or TRAF3-deficient T cells stimulated through IL-4R alone, consistent with our finding that TRAF3 deficiency does not affect IL-4R signaling without prior TCR stimulation (Figs. 5, *A* and *B* and 3, *C*–*F*).

TRAF3 constitutively associates with JAK1 in WT T cells and following type I IFN signaling (14) (Fig. 5, *A* and *C*). Interestingly, there was increased JAK1-associated TRAF3 in WT T cells stimulated through TCR–CD28 with or without subsequent engagement of IL-4R, compared with cells stimulated through IL-4R alone (Fig. 5, *A* and *C*). This may reflect increased sequestration of PTP1B by TRAF3, thereby enhancing IL-4-mediated STAT6 activation.

To test the prediction that PTP1B serves as a negative regulator of IL-4R-mediated STAT6 activation in T cells, we treated T cells with the selective PTP1B inhibitor TCS-401 (5, 34). PTP1B inhibition prior to stimulation with IL-4 increased the abundance of pSTAT6 Y641 in TRAF3-deficient T cells stimulated with IL-4 but did not affect IL-4-induced STAT6 activation in WT T cells (Fig. 5, *D* and *E*). This suggests that PTP1B is a negative regulator of IL-4R-induced STAT6 activation in T cells specifically in the absence of TRAF3.

To determine the impact of TRAF3-mediated regulation of PTP1B on the response of TCR–CD28-activated T cells to IL-4, we treated cells with TCS-401, then assessed activation of STAT6 following stimulation through TCR–CD28 alone, TCR–CD28 followed by IL-4, or IL-4 alone. Both WT and TRAF3-deficient T cells showed increased STAT6 activation when treated with TCS-401. TRAF3-deficient T cells that received TCR–CD28 stimulation followed by IL-4 had significantly more abundant pSTAT6 Y641 when PTP1B was inhibited with TCS-401 than when PTP1B was active (dimethyl sulfoxide control) (Fig. 5, *F* and *G*). In contrast to results discussed previously (Fig. 5, *D* and *E*), there was only a small impact of TCS-401 on IL-4R-mediated activation of STAT6 in TRAF3-deficient T cells in this experiment (Fig. 5, *F* and *G*), which may reflect variations in experimental setup.

**Figure 6:**
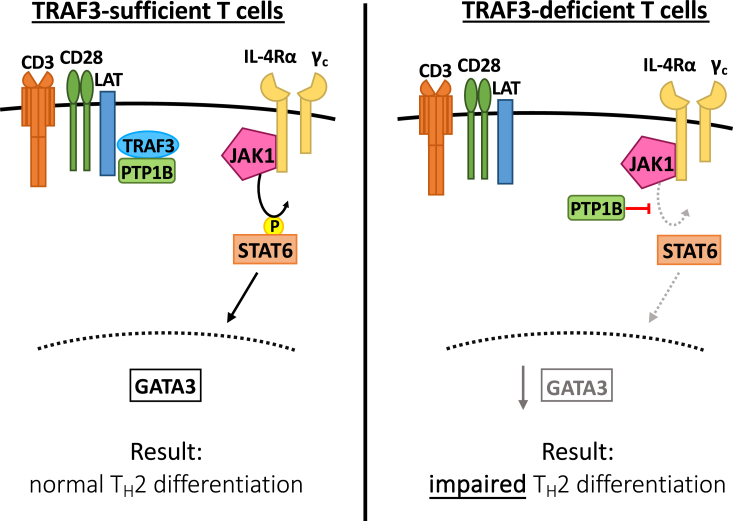
**Impact of TRAF3 on regulation of STAT6 activation mediated by TCR–CD28 and IL-4R signaling.** In TRAF3-sufficient T cells stimulated through TCR–CD28 (*left*), TRAF3 associated with the TCR–CD28–LAT complex interacts with PTP1B, preventing it from associating with the IL-4R complex. This allows IL-4R-mediated phosphorylation of STAT6 by JAK1 and downstream processes to proceed normally, resulting in normal GATA3 upregulation and T_H_2 polarization. In TRAF3-deficient T cells stimulated through TCR–CD28 (*right*), unrestrained PTP1B associates with the IL-4R complex. This impairs IL-4R-mediated phosphorylation of STAT6 by JAK1 and additional downstream processes, resulting in impaired GATA3 upregulation and reduced T_H_2 polarization. GATA3, GATA-binding protein 3; IL-4, interleukin 4; STAT6, signal transducer and activator of transcription 6; TCR, T-cell receptor; TRAF3, tumor necrosis factor receptor–associated factor 3.

Taken together, results presented here are consistent with a model whereby TRAF3 enhances crosstalk between the TCR–CD28 and IL-4R signaling pathways *via* restraint of the negative regulator PTP1B (Fig. 6).

## Discussion

A growing body of evidence demonstrates that TRAF3 is a multifaceted regulator of T-cell biology (reviewed in Refs. (2, 3)). Roles for TRAF3 are reported in regulation of each of the three signals required for T-cell activation: signaling through the TCR (4, 6), signaling mediated by costimulatory receptors (5, 11), and cytokine receptor signaling (10, 14). TRAF3 deficiency in T cells thus has a striking effect on T-cell development and *in vivo* immune responses. T-*Traf3*^−/−^ mice have altered proportions of CD4 and CD8 T-cell populations, defective cytokine production, and impaired *in vivo* responses to antigen (Ag) and pathogen challenge (6, 10, 14, 35). Humans with *TRAF3* haploinsufficiency have altered proportions of CD4 and CD8 T-cell populations, cytokine production, and homeostasis of T_reg_ and T_FH_ cells, which all contribute to the phenotype of immunodeficiency and autoimmunity (13). The mechanisms by which TRAF3 regulates the differentiation of CD4 T-cell subsets, and thus impacts *in vivo* immune responses, remain poorly understood.

The Akt–mTOR pathway plays a key role in CD4 T-cell fate decisions (18, 20, 36). Importantly, while this pathway is downstream of TCR–CD28-mediated signaling, which is defective in the absence of TRAF3 (4, 5, 6), the nature of the role of T-cell TRAF3 in Akt–mTOR signaling was not known prior to this study. Here, we showed that TCR–CD28-mediated phosphorylation of Akt at both T308 and S473 was reduced in TRAF3-deficient T cells. This is in contrast to the normal Akt phosphorylation observed in conventional CD4 T cells stimulated through IL-2R (10) and glucocorticoid-induced tumor necrosis factor receptor (11), in addition to the normal TCR–CD28-mediated phosphorylation and degradation of the distal signaling molecule IκBɑ^6^, once again demonstrating the cell type and receptor-specific context-dependent nature of the roles of TRAF3. This finding has implications for both Akt substrate specificity (21, 37) and signaling mediated by mTOR. mTOR signaling drives T-cell metabolic reprogramming, which in turn shapes T-cell fate and function (8). Investigation of the molecular mechanisms underlying TRAF3-mediated regulation of the mTOR pathway will provide further insight into how T-cell TRAF3 impacts multifaceted *in vivo* immune responses.

Differentiation of CD4 T-helper cell subsets is a complex process that is optimized by integration of multiple inputs, including TCR signal strength, Ag dose, costimulatory signaling, cytokine milieu, and metabolites (21, 38, 39, 40, 41, 42, 43). *In vitro* CD4 T-helper differentiation experiments cannot fully recapitulate *in vivo* requirements for CD4 T-cell fate decisions (44). However, these experiments enable both investigation of the signaling pathways underlying these fate decisions and the role of TRAF3 in regulation of these pathways. Additionally, TRAF3 regulates multiple types of receptors in T cells, making it challenging to identify the molecular mechanisms by which TRAF3 regulates specific individual receptor complexes in *in vivo* experiments. We thus turned first to *in vitro* models to identify the molecular mechanisms by which TRAF3 regulates activation of the transcription factors STAT1 and STAT6 to drive skewing toward the T_H_1 and T_H_2 fates. The TCR signal strength paradigm of CD4 T-helper subset differentiation (39, 45, 46) purports that high quality and quantity of TCR interactions with Ag-presenting cells promote skewing toward the T_H_1 fate, whereas weaker TCR signals promote skewing toward the T_H_2 fate. Additionally, the paradigm of T_H_1/T_H_2 crossregulation suggests that a failure to upregulate the key T_H_1 transcription factor T-bet pushes cells toward the default T_H_2 program (23, 24, 47). Unexpectedly, both T_H_1 and T_H_2 skewing were impaired in the absence of TRAF3 (Fig. 2). The impairment in T_H_1 skewing in TRAF3-deficient cells was not due to defective TCR–CD28-mediated STAT1 activation or early IFNɣR signaling (Fig. S1). It is possible that altered IL-12R-mediated activation of STAT4 contributes to the results presented here; however, we have not yet fully explored the impact of TRAF3 upon STAT4 activation due to limitations of commercially available reagents. Alternatively, our results may be explained by defective TCR-mediated signaling. T-*Traf3*^−/−^ mice have impaired development of iNKT cells due to a failure of TCR-mediated upregulation of T-bet (12). Notably, TCR signaling induces a T_H_1 molecular profile in a lymphocyte-specific kinase (Lck)–dependent manner (48); we previously reported a role for TRAF3 in regulation of Lck activation through interactions with the inhibitory kinase Csk (4).

There was minimal impact of the T_H_2-polarizing cytokine IL-4 when provided as a single stimulus on the skewing of TRAF3-deficient T cells toward the T_H_2 fate (Fig. 2). This finding, together with the precedent for regulation of cytokine receptor signaling by TRAF3 (10, 15, 30, 49), prompted us to investigate the role of TRAF3 in IL-4R signaling. We found that while TCR–CD28-mediated activation of STAT6 was reduced in TRAF3-deficient T cells (Fig. 3, *A*–*C*), early IL-4R-mediated signaling was intact (Fig. 3, *D*–*F*), suggesting that the observed defect was due to altered *integration* of TCR–CD28- and IL-4R-mediated signals. There is an established role for CD28-mediated signaling in enhancement of IL-4R signaling *via* increased sensitivity of IL-4R for IL-4 (50). Additionally, STAT6, a key mediator of GATA3 upregulation and skewing toward the T_H_2 fate, is recruited to CD28 and phosphorylated upon engagement of CD28 by its ligand (51). Engagement of CD28 primarily drives expression of the T_H_2 molecular profile *via* PKCθ (48), which is a target of mTORC2 (20, 52). Phosphorylation of Akt at S473, an indicator of mTORC2 activity, is reduced in TRAF3-deficient T cells stimulated through TCR–CD28 (Fig. 1, *A* and *C*). A possible explanation for the T_H_2 skewing defect presented here is that defective CD28-mediated signaling impairs the responsiveness of TRAF3-deficient T cells to IL-4, thereby impacting STAT6 activation and upregulation of GATA3 expression. The impact of TRAF3 upon CD28-mediated activation of PKCθ signaling is an intriguing question for future investigation.

TRAF3 regulates signaling mediated by multiple receptors *via* interactions with tyrosine phosphatases to modulate lymphocyte function. Interactions between TRAF3 and the tyrosine phosphatase PTPN22 restrain IL-6R signaling in B cells (49) and enhance TCR-mediated Lck activation and type I IFN signaling in T cells (4, 14). TRAF3 restrains IL-2R signaling in Treg through interactions with the phosphatase PTPN2 (10) and enhances TCR–CD28 signaling in conventional T cells *via* restraint of the phosphatase PTP1B (5). PTP1B has an established role as a negative regulator of multiple other signaling pathways, including TLR signaling in myeloid cells (53) and IL-4R signaling in B cells (27). Our results suggest that in T cells, TRAF3 enhances crosstalk between the TCR–CD28 and IL-4R signaling pathways *via* restraint of JAK1-associated PTP1B, thereby enhancing activation of STAT6 (Fig. 6). These results highlight a role for TRAF3 in regulation of signaling pathways that are important for T-cell fate decisions and offer a potential target for manipulation of T-cell fate and function to enhance *in vivo* immune responses. The present findings also add to our evolving understanding of how TRAF3 interacts with phosphatases to modulate signaling mediated by a variety of receptors. The impact of TRAF3 on the development of additional T-helper subsets, including T_H_17 cells and T_FH_ cells, and in regulation of key receptors involved in development of these subsets, is an area of ongoing investigation.

## Experimental procedures

### Mice

*Traf3*^*flox/flox*^ mice, backcrossed extensively to C57Bl/6 mice (54) and bred to *Cd4-*Cre mice to generate T-*Traf3*^*−/−*^ mice, were previously described (35, 54). A similar number of adult (2–6-month-old) male and female mice were used. Mice were age- and sex-matched for each experiment, and WT littermates were used as controls for all experiments. All mice were maintained under specific pathogen-free conditions at the University of Iowa and used in accordance with the National Institutes of Health guidelines under an animal protocol approved by the Institutional Animal Care and Use Committee at the University of Iowa.

### Mouse primary CD4^+^ T-cell isolation

Splenic CD4 T cells were isolated from adult mice with a negative selection kit (STEMCELL Technologies; catalog no.: 19851) according to the manufacturer’s protocol. Isolated cells were washed 1× with serum-free RPMI1640 (Life Technologies) prior to use.

### Cell lines and culture

HT28.11, a subclone of the human CD4 T-cell line HuT78 transfected to stably express CD28 (55), was a gift from Dr Arthur Weiss (University of California). The TRAF3^−/−^ subclone of HT28.11 cells, referred to here as crTRAF3^−/−^ or crT3^−/−^, was described previously (4). All T-cell lines were maintained in RPMI1640 supplemented with 10 μM B-mercaptoethanol (VWR International), 10% heat-inactivated fetal bovine serum (Life Technologies), 2 mM L-glutamine (Life Technologies), and 100 U/ml penicillin–streptomycin (Life Technologies).

### Antibodies and reagents

Abs used for immunoblotting included the following. Rabbit antibeta actin (catalog no.: 4967), rabbit anti–phospho Akt T308 (C31E5E; catalog no.: 2965), rabbit anti–phospho Akt S473 (D9E; catalog no.: 4060), rabbit anti-JAK1 (6G4; catalog no.: 3344), rabbit anti-PTP1B (catalog no.: 5311), rabbit anti–phosphoSTAT1 Y701 (58D6; catalog no.: 9167), rabbit anti-STAT1 (catalog no.: 9172), rabbit anti–phosphoSTAT6 Y641 (D8S9Y; catalog no.: 56554), and rabbit anti-STAT6 (D3H4; catalog no.: 5397) were purchased from Cell Signaling Technologies. Mouse anti-JAK1 (clone 73; catalog no.: 05-1554, used for immunoprecipitation (IP) of JAK1 from cell lysates) was purchased from MilliporeSigma. Rabbit anti-TRAF3 (H-122) was purchased from Santa Cruz Biotechnology. Stimulatory monoclonal antibodies (mAbs) against human and mouse CD3ε (clones OKT3 and 2C11, respectively) and CD28 (clones CD28.6 and 37.51, respectively) were purchased from eBioscience. Neutralizing mAbs against mouse IL-4 (clone 11B1; catalog no.: BE0045) and IFNɣ (clone XMG1.2; catalog no.: BE0055) were purchased from BioXCell.

The following carrier-free recombinant cytokines were purchased from BioLegend: human IL-2 (catalog no.: 589102), human IL-4 (catalog no.: 574002), mouse IL-4 (catalog no.: 574302), mouse IL-12 p70 (catalog no.: 577002), and mouse IFN (catalog no.: 575302). The PTP1B inhibitor TCS-401 (catalog no.: sc-204327) was purchased from Santa Cruz Biotechnology.

### *In vitro* T_H_1 and T_H_2 polarization

One day prior to splenic CD4 T-cell isolation, an appropriate number of wells in a 24-well plate were coated with 5 μg/ml of each ɑCD3ϵ (2C11; eBioscience) and ɑCD28 (37.51; eBioscience) mAbs diluted in 1× PBS (Life Technologies). The plate was incubated at 4 °C overnight, and wells were washed with 1× PBS before plating cells.

About 0.5 x 10^6^ cells/well were plated in 500 μl of complete RPMI1640 (supplemented with 10% heat-inactivated fetal bovine serum, 2 mM L-glutamine, and 100 U/ml penicillin–streptomycin; all purchased from Life Technologies). Polarization conditions were previously described (56, 57). Briefly, T_H_1 cells were differentiated in the presence of 2 ng/ml IL-2, 5 ng/ml IL-12, and 10 μg/ml ɑIL-4 Ab. T_H_2 cells were differentiated in the presence of 2 ng/ml IL-2, 10 ng/ml IL-4, and 10 μg/ml ɑIFNɣ Ab. In some experiments, cells were only cultured in the presence of plate-bound ɑCD3ε (2C11) and ɑCD28 (37.51) mAbs (eBioscience), without cytokine supplementation. Cells in all conditions were incubated at 37 °C with 5% CO_2_ for 72 h.

### Flow cytometry

Primary mouse CD4+ T cells were harvested after 72 h of culture, washed with serum-free RPMI1640, and divided evenly into the wells of a 96-well round bottom plate. FcɣR on the cells was blocked with 2% normal rat serum (STEMCELL Technologies) and 0.5 μg/ml ɑCD16/ɑCD32 Abs (eBioscience; catalog no.: 14-0161-81) in FACS buffer (1× PBS). (58) Cells were stained with fluorochrome-conjugated Abs against CD4 (GK1.5), CD11a (M17/4), CD44 (IM7), and CD90.2 (53–2.1) in the dark for 30 min on ice and washed with FACS buffer. For intracellular transcription factor staining, cells were fixed, permeabilized, and stained with the FoxP3/Transcription Factor Staining Kit (eBioscience; catalog no.: 00-5523), according to the manufacturer’s instructions. Cells were stained with fluorochrome-conjugated Abs against T-bet (eBio4B10), GATA3 (TWAJ), and their corresponding isotype controls. All fluorochrome-conjugated Abs were purchased from eBioscience. Data were acquired on an LSR II (BD) and analyzed with FlowJo software (FlowJo, LLC).

### PTP1B inhibition experiments

HT28.11 cells were resuspended in serum-free RPMI1640 medium and treated with 5.3 μM of TCS-401 or an equal volume of dimethyl sulfoxide for 2 h at 37 °C. Cells were washed 3× with serum-free RPMI1640, resuspended at a concentration of 0.5 × 10^6^ cells/200 μl, and stimulated with 10 μg/ml of each ɑCD3ε (OKT3; eBioscience) and ɑCD28 (28.6; eBioscience) mAbs for 2 h at 37 °C. Either TCR–CD28-activated or unactivated cells were subsequently stimulated with 20 ng/ml IL-4 for 15 min at 37 °C. Cell pellets were resuspended in 2× SDS-PAGE sample buffer (prepared as previously described (59)) and sonicated to prepare whole-cell lysates for Western blotting analysis.

### JAK1 IPs

About 10–15 x 10^6^ HT28.11 cells were stimulated with ɑCD3 and ɑCD28 Abs for 2 h and/or with IL-4 for 15 min as described previously. Cells were lysed with 200 μl of IP lysis buffer (0.5% Triton X-100, 100 mM NaCl, 40 mM Tris–HCl [pH 7.5], 1 mM MgCl_2_, 1 mM CaCl_2_, 2 mM Na_3_VO_4_, 0.05 mg/ml DNAse I [Roche], and EDTA-free mini-complete protease inhibitor tablet [Roche] on ice for 30 min). Lysates were incubated with ɑJAK1 Ab (clone 73; catalog no.: 05-1154; Millipore) at a concentration of 1:100 on a rotator at 4 °C overnight, then incubated with Protein G Dynabeads (Invitrogen) on a rotator at 4 °C for 1 h. Beads were washed 3× with IP lysis buffer, resuspended in 2× SDS-PAGE sample buffer, and boiled at 95 °C for 5 min to elute immunoprecipitated proteins.

### Western blot analysis

Proteins were separated by SDS-PAGE and transferred onto polyvinylidene difluoride membranes for Western blotting as previously described (4, 6, 35). Blots were imaged using Fujifilm LAS-4000, and densitometry was performed using Fujifilm Multi Gauge software (Fujifilm Life Sciences). Band intensity is reported as a ratio of raw densitometry values (protein of interest band intensity/loading control band intensity) or as “fold change to max,” for which all ratios are divided by the maximum value in that replicate, which is then set to 1. This allows pooling of replicates with similar relationships between different groups but wide variation in raw values.

### Statistical analysis

Results are presented as mean ± SEM of values obtained from multiple experiments, and graphs and statistical analyses were prepared using GraphPad Prism software (GraphPad Software, Inc). For experiments with primary mouse T cells, each “biological replicate” used cells from a different animal. For experiments with HT28.11 cell lines, “independent replicate” means experiments were not performed in parallel or using cells from the same passage/flask. Statistical tests are stated for each dataset in figure legends. Statistical significance was set as ∗*p* < 0.05, ∗∗*p* < 0.01, ∗∗∗*p* < 0.001, and ∗∗∗∗*p* < 0.0001.

## Data availability

All data necessary to evaluate the conclusions of this study are contained within the article or supporting information.

## Supporting information

This article contains supporting information.

## Conflict of interest

The authors declare that they have no conflicts of interest with the contents of this article.
