# Supplementary material for: TRAF3 regulates STAT6 activation and T-helper cell differentiation by modulating the phosphatase PTP1B
Source: J Biol Chem. 2024 Sep 2;300(10):107737. doi: 10.1016/j.jbc.2024.107737 (PMC11462019; doi:10.1016/j.jbc.2024.107737)
Supplement: Supporting information [file mmc1.pdf]

Supplemental figure 1

Primary mouse CD4 T cells

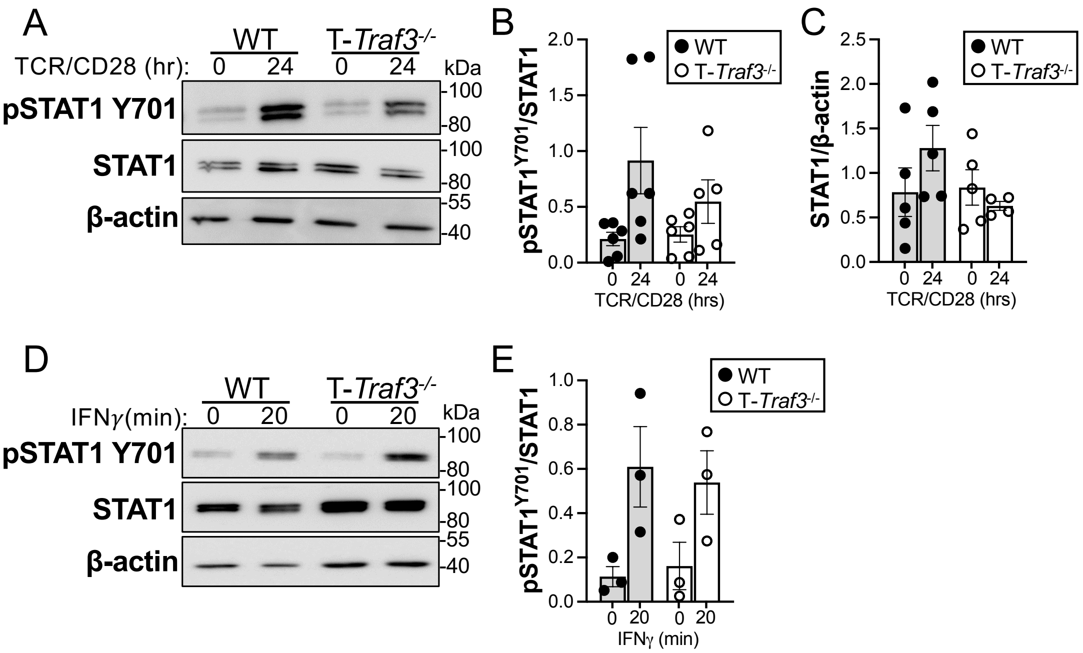

Supplemental Figure 1. Impact of TRAF3 on STAT1 activation in T cells.
Primary mouse CD4 T cells were stimulated as indicated, then lysed for quantification of proteins of interest. (A) Representative Western blot of phospho-STAT1 Y701 in mouse CD4 T cells ± 24hrs TCR/CD28 stimulation. (B) and (C) Quantification phospho-STAT1 Y701 (B) or total STAT1 (C) in five biological replicates, including the blots shown in (A). (D) Representative Western blot of phospho-STAT1 Y701 in mouse CD4 T cells ± 20min IFNy. (E) Quantification of three biological replicates, including blots shown in (F). Error bars represent mean ± SEM.
